# Supplementary figures and images for: BMP-Induced MicroRNA-101 Expression Regulates Vascular Smooth Muscle Cell Migration
Source: Int J Mol Sci. 2020 Jul 4;21(13):4764. doi: 10.3390/ijms21134764 (PMC7369869; doi:10.3390/ijms21134764)

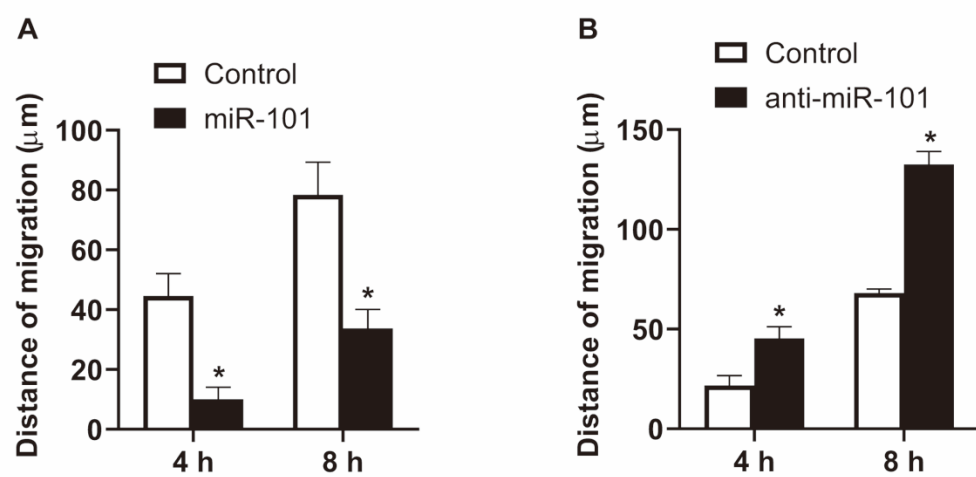

Supplementary Figure 1. miR-101 regulates VSMC migration.

Supplement: Supplementary file 1 [file ijms-21-04764-s001.pdf]
